# Health resources for South Africa: A scoping review

**DOI:** 10.4102/hsag.v25i0.1378

**Published:** 2020-07-29

**Authors:** Michelle Pascoe, Olebeng Mahura, Jessica Dean

**Affiliations:** 1 Department of Health and Rehabilitation Sciences/Child Language Africa, Faculty of Health Sciences, University of Cape Town, Cape Town, South Africa

**Keywords:** languages, cultures, healthcare, resources, cross-cultural

## Abstract

**Background:**

Healthcare is more effective when people are treated in their own language with respect for their culture. However, information about the availability and nature of health resources is fragmented and studies suggest few assessments, screening tools, or other health resources in many of South Africa’s languages.

**Aim:**

This scoping review identified health resources written in the eleven official languages of South Africa for health professionals to use for patient assessment and management.

**Meth ods:**

Databases were searched and information about resources collated and analysed.

**Results:**

Two-hundred-and-fifty two unique resources were found (444 items, if different language versions of the same resource were counted separately). All official languages were represented. The most widely used (excluding English) were Afrikaans (118 resources), IsiXhosa (80) and IsiZulu (55).

**Conclusion:**

Development of more health resources and critical evaluation of their validity and reliability remain important. This study contributes a preliminary database for South African health professionals, ultimately promoting improved service delivery.

## Introduction

Healthcare delivery should be equitable and accessible (United Nations 2018). One step towards this goal is by ensuring that professionals provide patient-centred care, with individuals’ culture and language considered (Marsiglia & Booth 2015; Truong, Paradies & Priest 2014; Wafula & Snipes 2014). Language is a human right but is often neglected and under-recognised (May 2011). In South Africa, the constitution specifies that all 11 official languages should have equal standing, but this ideal is not always realised in the public domain (Bornman et al. 2010; Southwood & Van Dulm 2015). The right to use and receive healthcare in one’s language must be incorporated into our healthcare systems if care is to be appropriate, fair and effective. Taking a patient’s language and culture into consideration can help reduce the over- and under-identification of difficulties (Southwood & Van Dulm 2015). This is especially important for those health professionals who rely primarily on their patient’s ability to understand and use language as part of the diagnostic and/or intervention process. Accurate diagnosis is more likely to lead to appropriately targeted interventions which will ultimately be more effective, reduce unnecessary financial burden and create positive attitudes to health services (Southwood & Van Dulm 2015).

This review focused on identifying health resources written in the 11 official languages of South Africa. The purpose of identifying the health resources was to find out what resources are available to health professionals to assist with patient management and treatment. Health resources were defined as tools used in the healthcare domain by health professionals for information gathering, diagnostic or intervention purposes. They include materials such as screening questionnaires, diagnostic assessments and intervention programmes, and may be electronic, paper-based or physical objects. Many studies suggest a paucity of assessments, screening tools and other health resources in the official South African languages (Bornman et al. 2010; Fetvadjiev et al. 2015).

One challenge of providing services that are linguistically and culturally appropriate arises when the resources used have not been specifically developed for the community being served: such resources may not yield valid findings (Gladstone et al. 2010). In the speech–language therapy domain, Barrat, Khoza-Shangase and Msimang (2012) found that translating tools such as the *Western Aphasia Battery* into languages spoken by patients was, for the most part, inappropriate, and Fetvadjiev et al. (2015) noted the rich diversity of languages and cultures in South Africa has been poorly accommodated in terms of psychological assessment, leading them to use an emic–etic approach in developing a culture-informed instrument for assessment of personality in South Africa. The translation of materials is a common practice for many clinicians in an attempt to ensure that clinical practice takes patients’ languages into account (Bornman et al. 2010; Southwood & Van Dulm 2015). However, translations often fail to target what the original tool aimed to assess. Translation from one language to another may not factor in cultural intricacies and may lead to the use of items or concepts that are unfamiliar to target populations (Trembath, Wales & Balandin 2005). Making use of a measure with a population besides the one it was created for, without taking any linguistic or cultural factors into consideration, puts the validity of such a measure at risk (Bornman et al. 2010).

Whilst it is widely acknowledged that there is a need for more locally relevant health resources and that it is a challenge to either adapt or develop such materials, information about the availability and quality of health resources in South Africa’s official languages remains fragmented and often inaccessible. Different health professions frequently develop tools in isolation without awareness of work that has been carried out in a related area or language (Rabin et al. 2015). Without shared information about resources – and the methodologies used to develop them – there may be redundancies and wasted resources instead of building on existing work. Although there are some resource listings for specific professions (e.g. see Mphahlele 2006 for an early published listing of speech–language therapy resources), there is no comprehensive, multidisciplinary health resources project focusing on South Africa. The lack of information and coherence may be because student projects and resources developed in clinical settings are often not published, and even when databases identify relevant papers, considerable work may still be required to identify the actual resource. Many research papers focus on specific theoretical questions, with the tools used in the study mentioned only tangentially, making it a time-consuming task to determine the nature of resources and one that must be performed manually.

A lack of culturally and linguistically appropriate health resources precludes the provision of quality healthcare services (Trembath et al. 2005). The use of such resources may improve the quality and access to healthcare (Truong et al. 2014). Health professionals need to be aware of current available resources in their contexts and have opportunities to build on the efforts of others and share their achievements and struggles. This study aims to contribute to this process by collating a database of health resources available in South Africa and describing these resources by language, health domain, nature of resource and targeted population.

## Methods

### Research design

This project used a scoping review following Arksey and O’Malley (2005). Scoping reviews aim to map crucial concepts underpinning a research area, the main sources and types of evidence available, and can be initiated as stand-alone projects particularly where an area is complex or has not been reviewed comprehensively before. The scoping review described in this article was the first step in an ongoing process of collating and reviewing resources. Our aim was not to investigate the reliability and validity of the available resources, but rather to produce a database with linguistically and culturally appropriate health resources for health professionals across a range of health disciplines. Arksey and O’Malley’s (2005) framework uses five main steps: (1) identifying the research question or aim. This review set out to describe health resources available in South Africa with a particular focus on the language of the resource, clinical domain, nature of material and the target group for whom it has been developed. (2) and (3) Identifying and selecting relevant studies. A search strategy, criteria for eligibility and study selection were devised. These are described in the following sections. (4) and (5) Data are then charted, collated and reported – the focus of the findings section of the article.

### Research team

The research team consisted of the three authors of this article together with a group of honours students, in consultation with a research librarian.

### Search strategy

We followed the three stages outlined by the Joanna Briggs Institute (Aromataris & Munn 2019). First, a pilot phase was initiated. Two databases (PubMed and CINAHL) were searched using a set of core terms. Titles, keywords and index terms taken from this initial set of papers were then used to develop our list of search terms, thus iteratively growing it. The pilot search was also used to check that the search process could be adhered to by all team members and troubleshooting was undertaken. Second, following the pilot phase, researchers then used the complete search term list with the full set of electronic databases. The databases included PubMed, CINAHL, EMBASE, PsycInfo, Scopus, Google scholar, EBSCOHost, ScienceDirect, Wiley Online Library, AccessMedicine, African Index Medicus Database and PsychNet. Given the scope of the search and the overlapping way in which the terms could be used, we kept the search terms broad in the initial search. The terms ‘health’, ‘resource’ and ‘South Africa’ were combined with MeSH terms and the Boolean operator ‘AND’. Wildcards of the following terms were used:

General terms: culture, language, assessment, evaluation, adaptation, translation.

Languages of interest: isiXhosa, isiZulu, Afrikaans, Siswati, Xitsonga, Sepedi, Sesotho, Setswana, Tshivenda, IsiNdebele, South African English.

Clinical areas: therapy, rehabilitation, primary healthcare, physiotherapy, psychology, family medicine, public health, obstetrics, geriatrics, paediatrics; neurology, psychiatry, orthopaedics, infectious diseases, HIV, respiratory medicine, pulmonology, TB, palliative care, ENT, occupational therapy, speech–language therapy, audiology, dietetics, nursing.

Health domains used in the search were adapted from the WHO Global Programme on Evidence for Health Policy Discussion Paper 43 (Sadana et al. 2002) which lists broad clinical domains. We did not select clinical areas in a purposeful fashion but rather set out to find all resources that met our criteria, aiming to maximise sensitivity and specificity in our search (Kovacs Burns et al. 2014). The third step was to use reference lists of the papers found to find further relevant papers. Grey literature searches were also completed using university repositories from South African universities, Google Scholar and feedback from a network of experts in the field. The search strategy was limited to English language articles. We did not include a cut-off date for the resources as we considered that a historical perspective on this body of work would be valuable. The search took place between February 2018 and November 2019. To ensure a valid and reliable process, measures were put in place. These included training sessions for the research team so that all researchers followed a standardised process in searching for and selecting resources, and entering the data; team briefings on a regular basis so that we could discuss any uncertainties and report on findings and challenges to date; and senior researchers (the authors of the article) verified all decisions and data collection from the other team members.

### Eligibility criteria

Studies were eligible if they described the translation, adaptation or development of health resources for South Africa. Our definition of health included a broad range of domains based on Sadana et al. (2002) which included quality of life, general health, language, child development, cognitive and executive functioning: hearing/auditory/vestibular, speech, literacy, participation/psycho-social aspects, motor abilities and outcomes, pain and mental health. Resources were defined as tools which are used in the healthcare domain by health professionals for information gathering, diagnostic or intervention purposes in clinical practice, that is materials such as screening questionnaires, diagnostic assessments and intervention programmes. Resources could be designed for use with individuals at any stage of the lifespan or carers/family reporting on other family members. We were interested in all resources used in the South African context and did not only consider tools that had been validated.

### Study selection

Each researcher screened the titles and abstracts of the articles from the electronic search. The research team then read full texts of papers that met the eligibility criteria. Papers were excluded when the eligibility criteria were not met and/or we could not obtain sufficient information about them that would enable other health professionals to access them. Resources (described within papers) had to have a title, be accessible (either through being shared in articles or projects) or through contacts with researchers or institutions. If a full-text article could not be accessed, there was no way of ensuring that any resources described fitted all the inclusion criteria and therefore such articles were not included in the database. For this study, we focused on the 11 official languages of South Africa, and therefore non-official South African languages or non-South African languages were excluded. We wanted to create an inventory of resources, rather than listing the journal papers or postgraduate projects themselves. In some cases, these were one and the same, but in other cases, the adaptation of a particular tool was a sub-component of a bigger research question making it a time-consuming task to determine the nature of resources and one that had to be performed through careful reading of full-text articles.

### Data collection

After identification of relevant papers and projects in the initial screening process, full-text articles or projects were assigned to individual researchers for reading and data extraction. Detailed information about resources was charted in a spreadsheet including the name of the resource, the official South African language/s in which the resource is available, the nature of the resource, the population for which the resource is designed, the health domain and then further information about how to access the resource whether through a published paper or postgraduate project. If a resource had been translated, the original authors were mentioned as well as the authors who developed the translated version. A training workshop was undertaken during the pilot phase to ensure that all data were entered in the correct format. To ensure reliable reporting, the senior researchers (authors of this article) also read and crosschecked all entries into the database; the third author was responsible for monitoring the entire database to ensure consistent formatting, accuracy and resolution of any queries. The percentage of agreement about study inclusion was approximately 96%. Differences were resolved via consensus between the three main researchers.

## Review findings

### Overview

**FIGURE 1:**
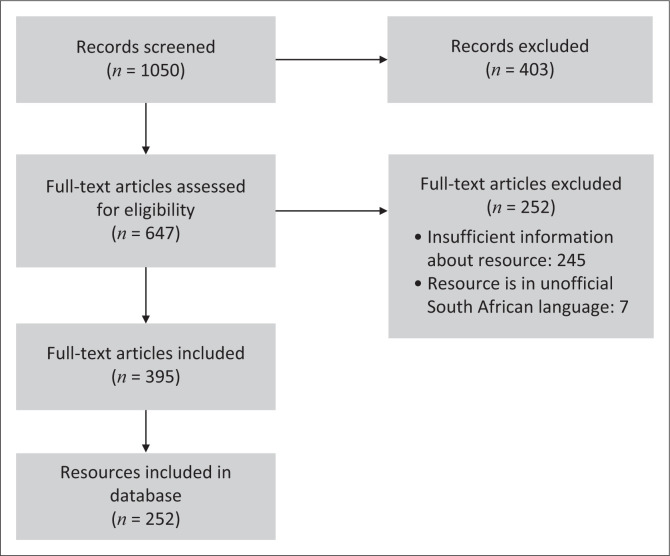
Flow diagram for paper selection process.

A total of 252 resources were included in the database of health resources for South Africa. In many cases, a resource had been adapted into multiple languages, and in such instances, these were collated into one entry, which detailed all the different language versions rather than having multiple entries. For example, The *South African Child Assessment Schedule* (SACAS; Barbarin, Richter & DeWet 2001) is available in Afrikaans, Sesotho and IsiZulu and these all appear as one entry, rather than as three separate entries. When all language versions of assessments were counted individually, a total of 444 resources were included in the database. Figure 1 provides an overview of the search process. It should be noted that our study was slightly different to most scoping reviews in that we aimed to list resources, which were located *through* the papers/projects found in our search, rather than simply listing the papers found. Figure 1 indicates that 395 articles were found and that these then led the research team to the 252 resources. Some of the resources were described in multiple articles, and in such cases, all relevant articles were cited in the entry for that resource.

**FIGURE 2:**
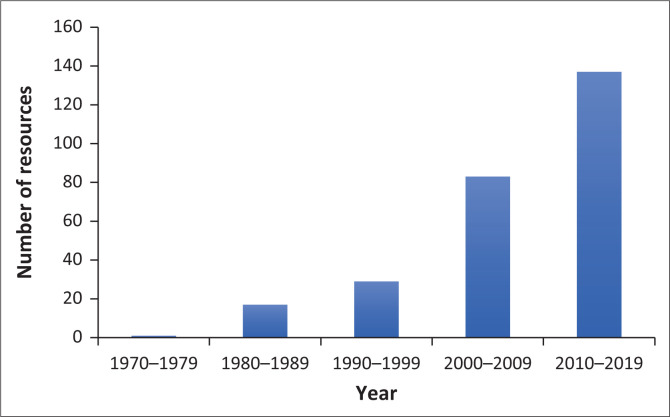
Number of health-related resources by year.

Figure 2 shows the growth in the number of available health resources for South Africa over the past 50 years. The number of resources has almost doubled each decade with the period 2010–2019 seeing the greatest number of new resources being made available, although the exponential growth from previous decades may have slowed down. Many of the projects prior to 1994 focused on Afrikaans. With the advent of the new constitution and formal recognition of more languages, many more projects focusing on a wider range of languages were noted. Most of the resources included in the database had been located through journal articles (*n* = 208) and those journal articles were cited as a way to access the tools or find out more about them. The growth in resources must also, to some extent, reflect the great increase in published papers over the last decades. The next largest group was that of postgraduate student projects, with 105 projects being cited in the database. Commercially available resources and ‘works in progress’ were also included in the database and comprised smaller groups. All of South Africa’s official languages were included in the database and the resources represented a wide variety of different health domains, types of resources and different populations, which will be described in greater detail in the section that follows.

### Languages of resources

**FIGURE 3:**
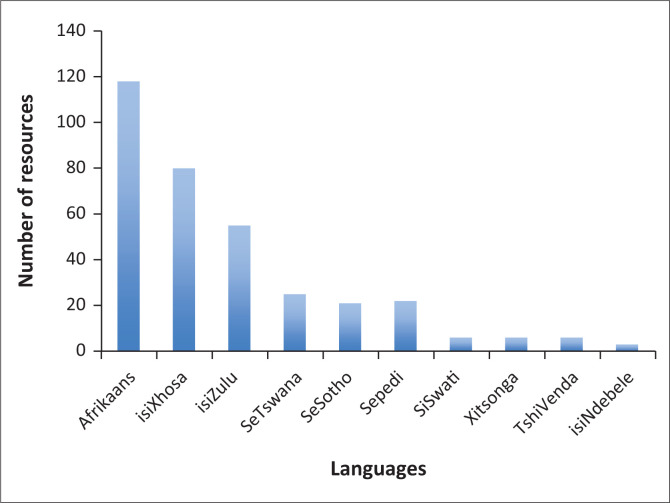
Distribution of resources by languages (Note: South African official languages, excluding English).

All 11 of South Africa’s official languages were represented in the database. The database includes South African English resources and there were some studies which had specifically developed resources in South African English. However, it was not always clear whether studies had specifically used South African English or if English from other countries had been adapted in significant ways. Given the relative abundance of health resources in English, we have excluded English studies from this section and focus instead on the other 10 official languages. Afrikaans, IsiXhosa and IsiZulu were the main languages represented in the database. Figure 3 shows the relative distribution of languages in the database. It is clear that some of the languages have very limited resources. For example, for IsiNdebele, we were only able to locate three resources: an adapted version of the *Intelligibility in Context Scale* (McLeod, Harrison & McCormack 2012; Pascoe & McLeod 2016); *Disruptive Behavior Disorder (DBD) Scales* (Meyer 2005) and the *South African Personality Scales* (Valchev et al. 2013). Siswati, Xitsonga and Tshivenda were all found to have six resources per language.

### Health domains

Eleven domains are represented in our data, as shown in Table 1, with the highest number of resources (61; 19.48%) relating to General health and Quality of life. There were also many resources in the clinical domains of Language (including expressive, receptive and pragmatic aspects of language) (57; 18.21%) and Mental Health and Personality (45; 14.38%). In many cases, resources were not assigned to only one category because some are intentionally broad and cover multiple domains. As we are wanting to establish a user-friendly and searchable database, we preferred to assign resources to multiple categories to facilitate users’ ability to find them.

**TABLE 1:** Overview of health resources by domain.

Domain	Number of resources in the database	Examples of resources
General health/quality of life	61	*EQ-5D* (Afrikaans, Sesotho, Setswana, isiXhosa, isiZulu; Jelsma and Ferguson, 2004; Scott, Ferguson and Jelsma, 2017)
Language (receptive, expressive and pragmatics)	57	*Expressive Vocabulary Test and Similarities test WISC-IV* (Afrikaans; de Sousa, 2016)
Mental health/personality	45	*Short moods and feelings questionnaire* (isiXhosa, English, Afrikaans; Rothon et al., 2011)
Child development	26	*Mullen Scales of Early Learning* (Afrikaans, South African English, Setswana, isiZulu; Bornman et al., 2010)
Literacy/preliteracy/phonological awareness	24	*Phonological awareness battery for isiXhosa* (Diemer, Van der Merwe and De Vos, 2015)
Speech production (articulation, phonology and intelligibility)	23	*Sepedi Test of Speech Intelligibility* (Sepedi; Fouche and Van der Merwe, 1999)
Motor abilities and outcomes	20	*Physical Activity Scale for the Elderly* (Setswana, isiZulu; Washburn et al., 1993)
Cognition and executive functioning	21	*Cognitive Linguistic Quick Test* (English, Sesotho, Setswana, isiZulu; Mupawose and Broom, 2010)
Hearing, auditory and vestibular	13	*Afrikaans Test for Sentence Recognition Thresholds in Noise* (Theunissen, Hanekom and Swanepoel, 2010)
Participation and psychosocial functioning	12	*International Classification of Functioning, Disability and Health (ICF) Checklist* (isiXhosa; Ka Toni, 2007)
Pain	11	*Brief Pain Inventory* (Afrikaans, Sepedi, Setswana, isiXhosa, isiZulu; Beck and Falkon, 2001; Parker, Jelsma and Stein, 2016)

### Nature of resources

Resources were classified into seven different resource types. The majority of resources were either classified as Questionnaires/Scales (106) or Diagnostic assessments (108). These terms were operationally defined as a paper or electronically based sheet of questions/statements for evaluation (Questionnaires/Scales) versus a clinician-administered tool/test that involved more than a sheet of questions/scales (Diagnostic assessments). Other categories included therapy/teaching materials (16), brochures/information booklets (17) and wordlists (16), all of which were less well represented. Again, there was some overlap between the nature of resources and in some cases, resources were included in multiple categories.

### Intended population for resource use

The resources which were analysed in our review were also classified by the population (age range) with which they were intended to be used. As with other areas of analysis, some resources fell into more than one category and could be used across a wide range of ages (e.g. children and adults). Almost half of the resources were designed for use with adults (129 resources, 47.7%). Eighty-six resources (31.85%) were found for direct use with children. A relatively small number were for caregivers to complete regarding their children (33; 12.22%) and for use with adolescents (22; 8.14%).

## Implications and recommendations

Given the large number of official languages and size of South Africa, there are relatively few health resources available for its people. English and Afrikaans resources predominate – unsurprising given the history of the country and the availability of English worldwide. IsiXhosa and IsiZulu resources were the next largest groups, fitting with their status as some of the most widely spoken languages in South Africa, although IsiZulu is reportedly home language to a larger proportion of the country than IsiXhosa (22.7% of the total population for IsiZulu in contrast to 16% for isiXhosa) (Government of South Africa 2017/2018). IsiXhosa is one of the main languages of the Western Cape (Government of South Africa 2017/2018) and although our search was national, our Cape Town-based team’s knowledge of local resources and contacts may have biased our results to some extent. Aside from the IsiXhosa/IsiZulu discrepancy, our findings otherwise reflected what is known about the number of first language speakers of each of the local languages. The very small number of IsiNdebele resources fits with it being the least widely spoken of all the official languages (Government of South Africa 2017/2018) so the call for such resources is likely to be less frequent. Nonetheless, the country’s constitution states that all languages should have parity of esteem and it is a fundamental human right for a person to receive healthcare through the medium of the home language. There is clearly a need for further development of resources in all of the indigenous languages and in particular in some of the languages that were not well represented in the study. The study revealed growing momentum in undertaking this work with over 100 resources being added to the database for the last 2 decades. This rapid increase reflects the country’s new constitution from 1994, which was associated with concerted efforts in promoting under-resourced languages from that time. There is still a great deal of work to be carried out in this regard given that Afrikaans (and English) still dominate, but clearly this has started to change in recent years.

The health domains that dominated in the database are inevitably those that need to be accessed through verbal means – such as measures of communication and cognitive processes. There were fewer measures in categories such as ‘motor activities and outcomes’ because many of these types of aspects can be objectively measured and observed without necessarily requiring language. General health and Quality of life studies predominated, reflecting the broad nature of this category, as well as the strong growth in quality of life as an outcome measure (Mercieca-Bebber et al. 2018). Speech and language therapy and associated domains of language (expressive, receptive and pragmatic aspects); speech (articulation, phonology and intelligibility); and literacy (including preliteracy and phonological awareness) were areas with a relatively large number of resources. This may be a reflection of the speech and language therapy profession’s focus on language, with adaptation and translation being areas of strength and interest for the professionals working in this area. Again, there may have been some bias here because this professional domain is the background of the research team. There were relatively few intervention resources, even for fields such as speech and language therapy, which is surprising given that this is the focus of that profession.

This review had several limitations. It was challenging to categorise many of the resources into only one category (for health domain, nature of resource and targeted group). Because we wanted to create a database that would be user-friendly and helpful to researchers and clinicians when searching for a health resource, we opted to assign multiple categories when this was indicated. This created some challenges for describing the results of the scoping review but was judged as important for the practical value of the resulting database. Our vision was to support health professionals in a practical way by making it easy to find the tools they need. This study took place over a limited time period (19 months) and although we aimed to access all relevant resources, the process was time-consuming and labour-intensive and we cannot claim that it is comprehensive. The scoping review presents a static set of results but the development of the database will be ongoing to ensure that we include further resources as these become available. There are also likely to be many clinicians who have developed tools for use in their clinical settings that have not been published and would not be accessible using our search strategy. Future plans for this project are to make the database available online and to invite participation and submission of resources from a wider group of stakeholders.

This study did not involve quality evaluation of resources and further projects would need to be undertaken to share information about the psychometric properties and validity and reliability of assessment instruments. Facilitation of an online community of practice is a way of empowering those working in this field to move away from a dependency on western tools and grow local expertise. The influence of information technology on health and social well-being has been documented both in developed and developing contexts (Pimmer et al. 2014). Our own experience of online media for support, promoting social belonging and building community encourages us to develop this line of work.

## Conclusion

The scoping review identified over 400 health resources written in the 11 official languages of South Africa for health professionals to use for patient assessment and management. All official languages were represented. The most widely used (excluding English) were Afrikaans (107 resources), IsiXhosa (71) and IsiZulu (49). There is a need for further development of resources in all of the indigenous languages and in particular in some of the languages that were not well represented in the study, such as isiNdebele. Despite its limitations, this work contributes to providing health workers and researchers with better access to more resources in all the local languages, with better links between the different disciplines, and examples of best practice in cross-cultural adaptation and translation. This will ultimately serve to improve health services as well as create opportunities for addressing theoretical questions at the interface of linguistics, information sciences and healthcare.
